# Supplementary figures and images for: Deep learning based predictive modeling to screen natural compounds against TNF-alpha for the potential management of rheumatoid arthritis: Virtual screening to comprehensive in silico investigation
Source: PLoS One. 2024 Dec 5;19(12):e0303954. doi: 10.1371/journal.pone.0303954 (PMC11620472; doi:10.1371/journal.pone.0303954)

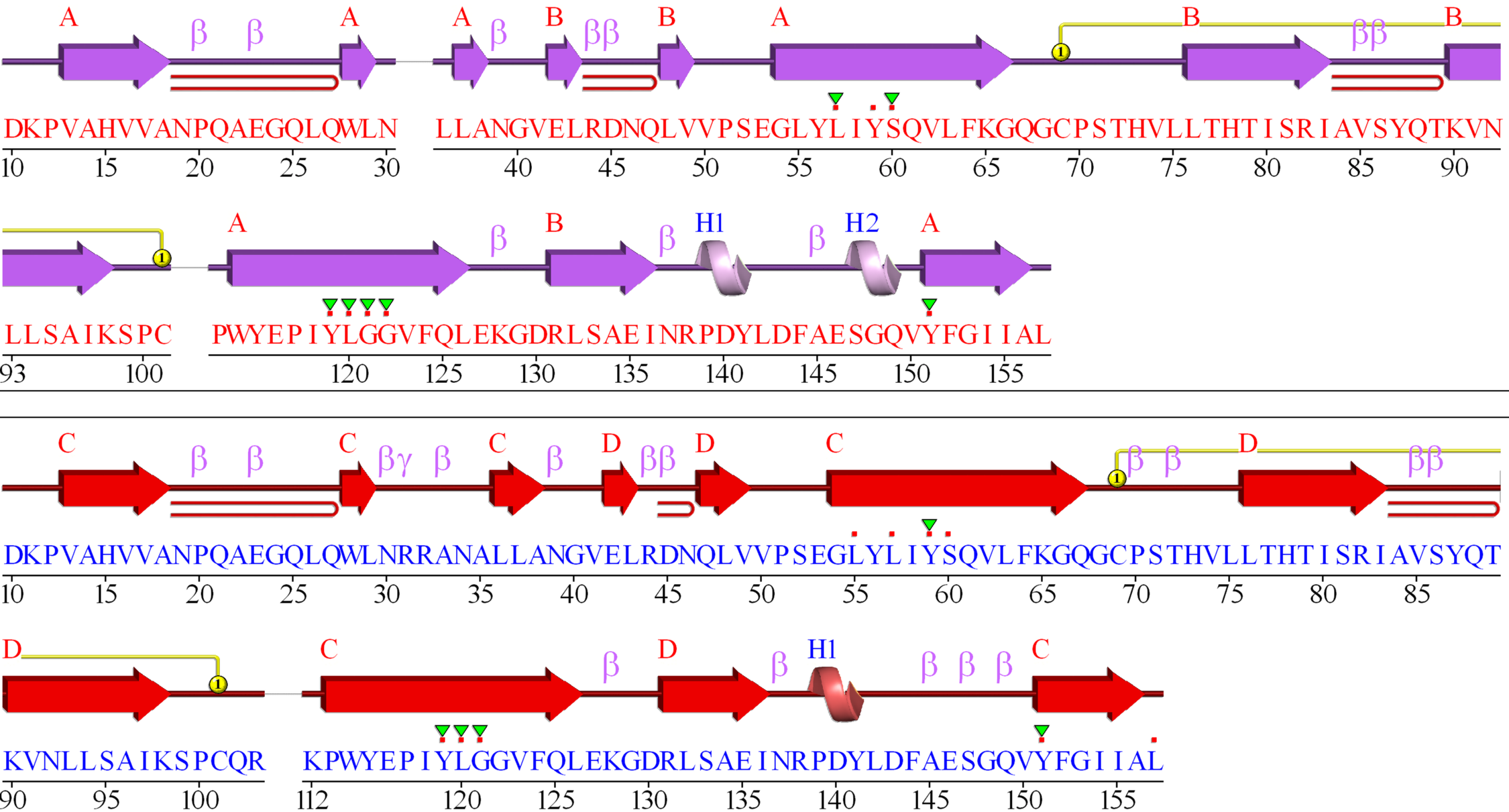

Supplement: S1 Fig — (TIFF) [file pone.0303954.s001.tiff]

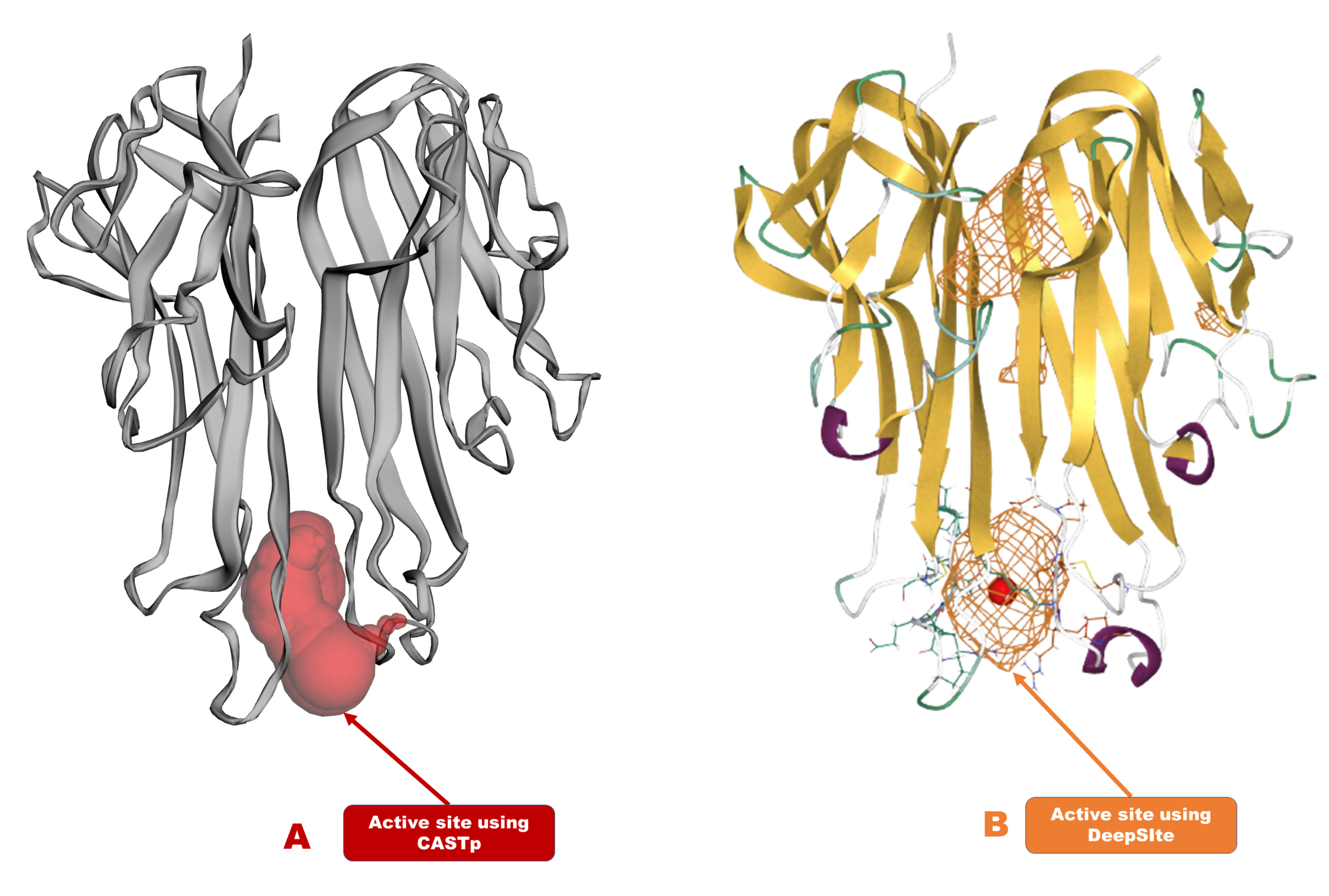

Supplement: S2 Fig — (TIFF) [file pone.0303954.s002.tiff]
