# Supplementary material for: Deep learning based predictive modeling to screen natural compounds against TNF-alpha for the potential management of rheumatoid arthritis: Virtual screening to comprehensive in silico investigation
Source: PLoS One. 2024 Dec 5;19(12):e0303954. doi: 10.1371/journal.pone.0303954 (PMC11620472; doi:10.1371/journal.pone.0303954)
Supplement: S1 Table — (DOCX) [file pone.0303954.s003.docx]

**S1 Table. Missing Amino acid residues among 4 chains of native protein (TNF-α) and their position range.**

| **Chains** | **Number of Missing Residues** | **Segment ranges** |
| --- | --- | --- |
| **A** | 16 | 31-35, 102-112 |
| **B** | 8 | 104-111 |
| **C** | 19 | 31-35, 86-88,102-112 |
| **D** | 7 | 104-110 |
