# Supplementary material for: Deep learning based predictive modeling to screen natural compounds against TNF-alpha for the potential management of rheumatoid arthritis: Virtual screening to comprehensive in silico investigation
Source: PLoS One. 2024 Dec 5;19(12):e0303954. doi: 10.1371/journal.pone.0303954 (PMC11620472; doi:10.1371/journal.pone.0303954)
Supplement: S2 Table — (DOCX) [file pone.0303954.s004.docx]

**S2 Table. Characterization of protein active sites using CASTp server.**

| **Pocket ID** | **Area** Å**^2^** | **Volume** Å**^3^** | **Residues** |
| --- | --- | --- | --- |
| 1 | 293.172 | 257.80 | Chain A – GLY59, CYS60, PRO91, CYS92, GLN93,ARG94, GLU95, THR96, PRO97, ALA100, GLU101, LYS103, PRO104, TRP105 |
|  |  |  | Chain B – PRO61, SER62, HIS64, SER90, PRO91, CYS92, GLN93, ARG94, GLU95 |
