# Supplementary material for: Deep learning based predictive modeling to screen natural compounds against TNF-alpha for the potential management of rheumatoid arthritis: Virtual screening to comprehensive in silico investigation
Source: PLoS One. 2024 Dec 5;19(12):e0303954. doi: 10.1371/journal.pone.0303954 (PMC11620472; doi:10.1371/journal.pone.0303954)
Supplement: S3 Table — (DOCX) [file pone.0303954.s005.docx]

**S3 Table. Characterization of protein active sites using Deep Site server.**

| **Pocket ID** | **Score** | **Residues** |
| --- | --- | --- |
| 1 | 0.99 | Chain A - PRO91, CYS92, GLN93, ARG94, GLU95, THR96, PRO97, ALA100, GLY99, ALA102, GLU101, LYS103 |
|  |  | Chain B – SER90, PRO91, CYS92, GLN93, ARG94, GLU95, LEU66 |
