# Supplementary material for: Deep learning based predictive modeling to screen natural compounds against TNF-alpha for the potential management of rheumatoid arthritis: Virtual screening to comprehensive in silico investigation
Source: PLoS One. 2024 Dec 5;19(12):e0303954. doi: 10.1371/journal.pone.0303954 (PMC11620472; doi:10.1371/journal.pone.0303954)
Supplement: S1 File — (DOCX) [file pone.0303954.s007.docx]

>Chain A
DKPVAHVVANPQAEGQLQWLNRRANALLANGVELRDNQLVVPSEGLYLIYSQVLFKGQGCPSTHVLLTHTISRIAVSYQTKVNLLSAIKSPCQRETPEGAEAKPWYEPIYLGGVFQLEKGDRLSAEINRPDYLDFAESGQVYFGIIAL

>Chain B
DKPVAHVVANPQAEGQLQWLNRRANALLANGVELRDNQLVVPSEGLYLIYSQVLFKGQGCPSTHVLLTHTISRIAVSYQTKVNLLSAIKSPCQRETPEGAEAKPWYEPIYLGGVFQLEKGDRLSAEINRPDYLDFAESGQVYFGIIAL
